# An Eye-Tracking Study of Multiple Feature Value Category Structure Learning: The Role of Unique Features

**DOI:** 10.1371/journal.pone.0135729

**Published:** 2015-08-14

**Authors:** Zhiya Liu, Xiaohong Song, Carol A. Seger

**Affiliations:** 1 School of Psychology, Center for Studies of Psychological Application, South China Normal University, Guangzhou, China; 2 Cognitive Psychology, Molecular, Cellular, and Integrative Neurosciences, Colorado State University, Fort Collins, Colorado, United States of America; Bournemouth University, UNITED KINGDOM

## Abstract

We examined whether the degree to which a feature is uniquely characteristic of a category can affect categorization above and beyond the typicality of the feature. We developed a multiple feature value category structure with different dimensions within which feature uniqueness and typicality could be manipulated independently. Using eye tracking, we found that the highest attentional weighting (operationalized as number of fixations, mean fixation time, and the first fixation of the trial) was given to a dimension that included a feature that was both unique and highly typical of the category. Dimensions that included features that were highly typical but not unique, or were unique but not highly typical, received less attention. A dimension with neither a unique nor a highly typical feature received least attention. On the basis of these results we hypothesized that subjects categorized via a rule learning procedure in which they performed an ordered evaluation of dimensions, beginning with unique and strongly typical dimensions, and in which earlier dimensions received higher weighting in the decision. This hypothesis accounted for performance on transfer stimuli better than simple implementations of two other common theories of category learning, exemplar models and prototype models, in which all dimensions were evaluated in parallel and received equal weighting.

## Introduction

Effective behavior in the human environment requires the ability to form categories of objects, people, and events and to link these categories with appropriate responses. Category members differ along many dimensions and on many specific features. This study focuses on the property of feature uniqueness and argues that it may play a very important role in category representation. A unique feature is a feature that appears in only one category; it can be but need not be a typical feature of the category. For example, in comparing equines and raptors, if a zebra and an owl happen to be exemplars of corresponding categories, the stripes of the zebra and the catlike head of the owl are unique, though not typical features of their corresponding category.

Unique features have received relatively little attention, likely due to limitations of standard task design. In a typical category learning task in which discrete features are manipulated, exemplars are formed by choosing one of two (or more) possible features within each feature dimension. For example, imagine a category structure with 4 dimensions, possibly simulated extraterrestrial animals which can vary in terms of their head, feet, hand, and body shape. The possible values within each dimension (e.g., the head) can be denoted as *1*, *2*, or *3* (e.g., *1* could denote a triangular head, *2* could denote a square head, and *3* could denote a circular head). Thus, the configuration A1 (*2 1 3 1*) might describe a particular exemplar with different feature values across the four dimensions. The subject’s task is to decide the most appropriate category membership for the stimulus. Many category learning tasks include categories with only two feature values (e.g., round or triangular) in each dimension (e.g., head shape) and are therefore unable to examine the effect of feature uniqueness on learning, because if there are only two feature values, features can be unique only if they are also deterministic. Family resemblance category structures (such as the classic Medin and Schaffer “5–4” category learning task) [1–5] and linearly and nonlinearly separable categories [6–7] typically adopt two feature values within each dimension. Markman and Maddox (2003) utilized multiple feature values within dimensions, but did not examine the effects of having a unique feature [8].

This study used a category structure (see Table 1) with 4 dimensions, each of which included 3 potential feature values. Dimensions 2 (D2) and 3 (D3) included a unique feature, defined as one that appears in only one category, that when present could be used to categorize an exemplar with perfect accuracy. For D2 and D3, the unique features were feature 1 for category A, and feature 2 for category B. It is important to note that a unique feature is not always a prototypical feature for the category; unique features can be relatively rare yet diagnostic, as in D2: the unique feature only appears twice out of the five stimuli from each category. We also distinguished between strong and weak prototypical features: a strong prototypical feature is not only the most typical, but has a relatively high frequency providing substantial probabilistic evidence of category membership (in our category structure, the strong prototypical features occurred on 3 of the 5 category exemplars); a weak prototypical feature is still the most typical, but the overall frequency is lower (in our category structure, the weak prototypical features occurred in 2 of the 5 category exemplars). As shown in Table 1, values *1* and *2* for D3 are both unique (characteristic of only one of the categories) and strongly prototypical (characteristic of 3/5 of the stimuli). D2 included a unique feature that was only weakly prototypical: feature 1 is unique to category A, and feature 2 to category B, but both are characteristic of only 2/5 of the stimuli. Dimension 1 (D1) included strong prototypical features, for example, feature 1 in category A which occurred in 3/5 of the stimuli, but no feature was unique to either category. Dimension 4 (D4) included neither unique features, nor strong prototypical features. In summary, we used four kinds of features across 4 dimensions: D3 included features that were both unique and strongly prototypical; D1 included only strong prototypical features; D2 included features that were unique and weakly prototypical; D4 included neither strongly prototypical nor unique features.

**Table 1:** The multiple-value-feature category structure in the experiment.

Learning phase stimuli	Dimension			
	D1	D2	D3	D4
Mean cue validity	.60	.70	.80	.53
Category A				
A1	2	1	3	1
A2	1	3	3	1
A3	1	3	1	2
A4	1	3	1	3
A5	3	1	1	3
Category B				
B1	1	3	2	2
B2	2	3	2	2
B3	2	2	3	3
B4	3	2	3	1
B5	2	3	2	3
Transfer phase stimuli				
T1	1	2	1	2
T2	1	1	2	2
T3	2	2	1	3
T4	2	1	2	1

*Note*. A1~A5 are the exemplars of category A, and B1~B5 are the exemplars of category B. These exemplars are obtained from two prototypes, A0 (*1311*) and B0 (*2322*). D1~D4 are assigned to head, wings, tail and feet across subjects using a Latin Square. D3 is referred to as the unique plus prototypical dimension: for category A the unique value is 1, and for category B the unique value is 2. Only category A exemplars can have the value 1, and only B exemplars can have the value 2. However, it is also possible for either category to have the neutral feature value 3, which is equally diagnostic of both categories. D3 has a feature that is both unique and strongly prototypical. D1 is includes a strongly prototypical feature, with 1 the prototypical value for category A, and 2 the prototypical value for B. D2 is designed to be a unique but only weakly prototypical dimension, with 1 the unique value for category A, and 2 the unique value for B. Dimension 4 includes only weakly prototypical features. Cue validity for each dimension was calculated as the average proportion of stimuli in which the feature present correctly indicate category membership. In all cases, feature value 3 were .5; for unique features values were 1.0, and for prototypical features values were based on relative frequency in each category; for example, in Dimension 1, feature 1 has a .75 validity for category A and a .25 validity for category B. There are four pairs of similar exemplar between leaning and transfer items, T1 (*1212*) & A3 (*1312*), T2 (*1122*) & B1 (*1322*), T3 (*2213*) & B3 (*2233*), and T4 (*2121*) & A1 (*2131*), each pair has three overlap features. Our dimensional search hypothesis predicts that the four transfer items, T1, T2, T3, and T4, will be classified as A in a probability sequence as T1>T3>T2>T4. At the same time, Prototype theory (PT) and Exemplar theory (ET) will give the others predictions on these four items. Because T1 has two features overlap with the A0 (*1*,*3*,*1*,*1*) but one with B0 (*2*,*3*,*2*,*2*), T2 has two features overlap with the B0 but one with A0, and T3 and T4 each has only one feature overlap with A0 and B0, PT predicts that the A probability sequence will be T1>T4 = T3>T2; Because T1 and T4 are similar with one of the A category items, while T2 and T3 are similar with one of the B category items, ET predict that the sequence will be T1 = T4>T2 = T3.

In this paper we further considered how rule based learning might occur within a category structure with discrete dimensions. We hypothesized that subjects would evaluate the dimensions in order of utility, which would be learned during trial and error category learning, and that this order of evaluation would be reflected in eyetracking measures, including overall number of fixations, length of average fixation, and initial eye fixation within a trial. Meier and Blair [9] showed that subjects showed a bias towards optimizing eye fixations for the most efficient categorization strategy (fewest total number of movements) even at the cost of evaluating some features with lower overall probabilistic information about category membership. In the context of our task, we hypothesized that unique and strongly typical features would receive higher weighting than other features. Specifically, we predicted that D3 should be the highest weighted dimension because the unique and strongly typical features (1 in Category A, 2 in Category B) provide substantial evidence for category membership; this feature alone can give the correct category for 60% of the stimuli. At the other extreme, we predicted that D4 should be the lowest weighted dimension because it has no unique features and only weakly typical features. Dimensions 1 and 2 allow us to directly compare strongly prototypical but not unique with unique but weakly prototypical features. We did not make strong predictions as to which would be most important, since both possibilities are plausible. The strongly prototypical dimension D1 could receive higher attention because useful information is available on a higher proportion of stimuli (6 / 10, versus only 4 / 10 in D2). On the other hand, when the unique feature is present in D2 it is more useful for categorization, as reflected in the higher mean validity for D2 across stimuli (see Table 1 for cue validity calculations). All together, we then predicted that the order of importance and hence evaluation would be [D3, D1 or D2, D4]. In this study, we used eye tracking as well as accuracy as dependent measures of learning. We take eye movement as a proxy for attention [5, 10]. Using eye tracking, we examined the weighting of different dimensions in terms of total observation time and number of fixations. We predicted that these measures would reveal highest attentional weighting for the dimensions in the order stated above.

We furthermore examined whether other standard theories of categorization could account for our results. There are three major theories or models of how categories are represented: rule-based, prototype-based, and exemplar-based. Rule-based theories assume that category learning is a process of discovering an explicit rule to maximize accuracy [11–13]. Dimensional search is a type of rule-based theories, as examined in more depth in the discussion. Prototype-based theories assume that stimuli are categorized on the basis of their similarity to category prototypes stored in memory [14–16]. A category prototype is generally defined as the average or most typical member of a category. Exemplar-based theories assume that the categorization of a new exemplar is based on its similarity to the representations of all previously encountered exemplars stored in memory [1, 2, 17]. A fundamental difference between dimensional sort and prototype and exemplar theories is that the former can account for differential reliance on a subset of dimensions or features, whereas both prototype and exemplar theories posit that all features are evaluated and contribute to the final decision. We utilized transfer stimuli that differ in the predictions made by dimensional sort, exemplar theory and prototype theory.

Finally, for purposes of comparison with previous studies by Rehder and Hoffman [5], we also fit two models to the eye-tracking learning data, the generalized context model (GCM) and multiplicative prototype model (MPM), which are well-established models of exemplar learning and prototype learning, respectively. Rehder and Hoffman [5] conducted an eye-tracking study to test which model best fit attention allocation when learning the Medin and Schaffer 5–4 category structure [1, 5]. They found that GCM better accounted for their results than the MPM. This study adapted their eye-tracking and mathematical modeling methods to examine whether GCM (ET) or MPM (PT) provides the best account of learning in our task.

## Method

### Subjects

The Institutional Review Board of South China Normal University approved this study. All subjects provided their written and verbal consent to participate in this study. The subjects were 42 undergraduate students at South China Normal University who were paid for their participation. All had normal vision or better with corrective lenses. For the primary eye tracking analyses we removed data for two subjects because of an excessive number of eye-tracking failures (greater than 10% of trials) in which the eye tracker could not locate the subjects’ pupil position (e.g., because of blinks). These subjects were retained for behavioral and individual differences analyses.

### Materials

A multiple-value-feature category structure (see Table 1) was developed for this study. There were two categories, A and B; A1~A5 are the trained exemplars of category A, and B1~B5 are the exemplars of category B. Exemplars of each category were obtained from two prototypes, A0 (*1311*) and B0 (*2322*), respectively. D1, D2, D3, and D4 are the four dimensions that each could be assigned to the head, wings, tail, and feet. In addition, four new stimuli were presented only in the transfer phase (T1, T2, T3, and T4), designed to allow us to discriminate between exemplar, prototype, and dimension sort strategies.

**Fig 1:**
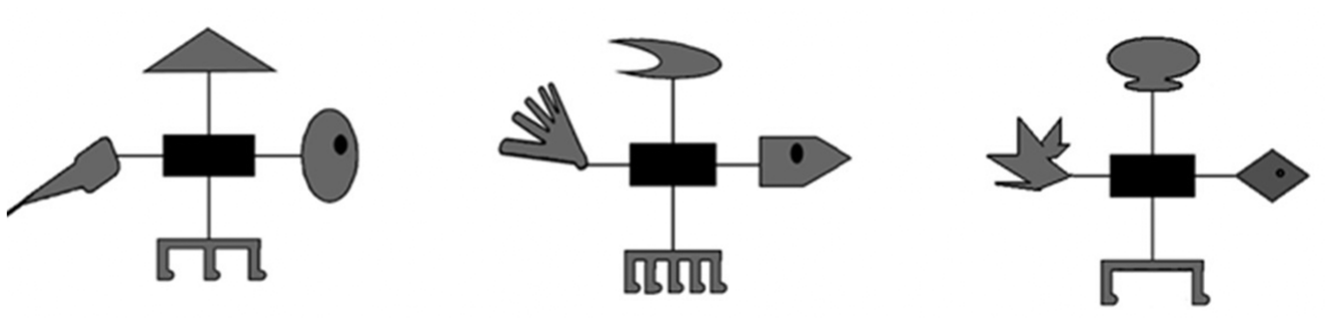
Stimulus features used in the study. The leftmost stimulus illustrates one possible assignment for the features (*1111*); the middle stimulus for the features (*2222*) and rightmost stimulus for the three remaining features (*3333*). Assignment of specific features to abstract roles within each dimension was randomized and counterbalanced across participants.

The prototypical stimuli are shown in Fig 1 (similar to Rehder and Hoffman, [5]). The center of each insect was a black rectangular body from where extended four black lines to four body parts instantiating the four dimensions. Each dimension had three main values, as illustrated in Fig 1, the head: oval, pentagon, and diamond; wings: parachute, wing, and balloon; tail: stinger, mallow, and maple; and feet: two, three, and five. A Latin square was used to assign physical dimensions to abstract dimensions so that each physical dimension value (e.g., the 3 head shapes) and feature role (e.g., 1, 2, and 3 for each stimulus in Table 1) was paired equally often across subjects. This balancing was essential, as subjects showed a tendency to look at heads more often than other body parts, and that could have been confused with the desired signal otherwise.

### Procedure

The subjects were seated at a personal computer with a color screen. They were told that they would see a series of drawings of bugs and asked to classify them into one of two mutually exclusive categories. The experiment consisted of three phases: a learning phase, a filler phase, and a transfer phase. In the learning phase, subjects learned the category via trial and error and continued until they performed three consecutive blocks with a combined accuracy of at least 90% or until they completed a maximum of 28 blocks (280 trials). After achieving criterion, the categorization task was terminated. In each block (10 trials), the order of stimulus presentation was determined randomly. Each learning trial began with a drift correction in which the subject fixated on a small cross at the center of the screen. The subjects classified the exemplar as belonging to either the “A” or ‘‘B” category at their own pace by pressing the “F” and “J” keys on the keyboard, respectively. The assignment of categories to the A or B labels was counterbalanced across subjects. After their response, the subjects were told whether they were correct, and the correct category label and the feedback (correct or incorrect) remained on the screen for 4 s. After the learning trials, there was a brief filler task in which they were asked to solve 10 simple arithmetic problems. Then all the subjects carried out the transfer task, in which they categorized four new stimuli (see Table 1). The task was programmed using the E-Prime software (Psychology Software Tools, Pittsburgh, Pennsylvania), version 1.2.

### Eye-tracking dependent variables

We recorded from a single eye with the Eyelink 1000 (Canada) eye-tracking system. The visual angle in width and in height of the entire stimulus was approximately 15°. Four areas of interest (AOIs) were defined as polygons encompassing the physical location of each of the four features on the computer screen; each was approximately 6° in width and height. All fixations outside of those AOIs were removed, as were any fixations that occurred after the subjects made their response. Several dependent variables were derived from the eyetracking data. The *number of fixations* is the average number of times a dimension is fixated on during a trial. The *fixation time* is the total number of milliseconds that a dimension is fixated on during a trial. *Mean fixation duration* is the average number of milliseconds that the dimension was fixated across all fixations within the trial. We also calculated the relative proportion of fixations for each dimension, and the probability that each dimension was fixated at least once during a trial. Finally, we identified the first fixation of each trial and calculated the relative frequency of first fixation for each dimension.

## Results

On average, subjects required 12 blocks (*Mean* = 11.67, *SD* = 6.67, range 3–28) to reach criterion.

Our first eyetracking analyses examined fixations overall for each stimulus dimension across training in order to establish if subjects preferentially viewed any of the dimensions, and if so, whether the patterns of fixations were consistent with our predictions. Table 2 shows the mean total fixation time, number of fixations, and mean fixation duration for each dimension across all training trials and subjects. A test of within-subjects effects showed that the total fixation time and number of fixations on the four dimensions were significantly different [*F* (3,37) = 17.78, *p*<.001; *F* (3,37) = 50.25, *p* < .001, respectively]; [mean fixation duration: not significantly different, *F*(3,37) = 1.62, *p*>.05]; pairwise comparisons showed that the up-to-down sequences of dimensions were D3>D1 = D2>D4 for fixation time and number of fixations. These results indicated that the most attention was devoted to D3, and least to D4, with D2 and D1 intermediate. It should be noted that fixation number and fixation time are not independent; the greatest predictor of fixation time is the number of fixations [18]. As shown in Table 2, when mean fixation duration was calculated (as total fixation time divided by number of fixations) individual fixation lengths did not differ significantly. Therefore, in subsequent analyses we focused on measures based on number of fixation and order of fixation.

**Table 2:** Fixation time and fixations of a trial on different dimensions.

	Total Fixationtime (*ms*)		Number of Fixations		Mean Fixation duration (*ms*)	
	*Mean*	*SD*	*Mean*	*SD*	*Mean*	*SD*
D1	408	446	1.39	1.16	253	196
D2	366	340	1.53	1.09	227	121
D3	742	675	2.79	1.61	264	161
D4	138	172	0.569	0.69	229	135

**Fig 2:**
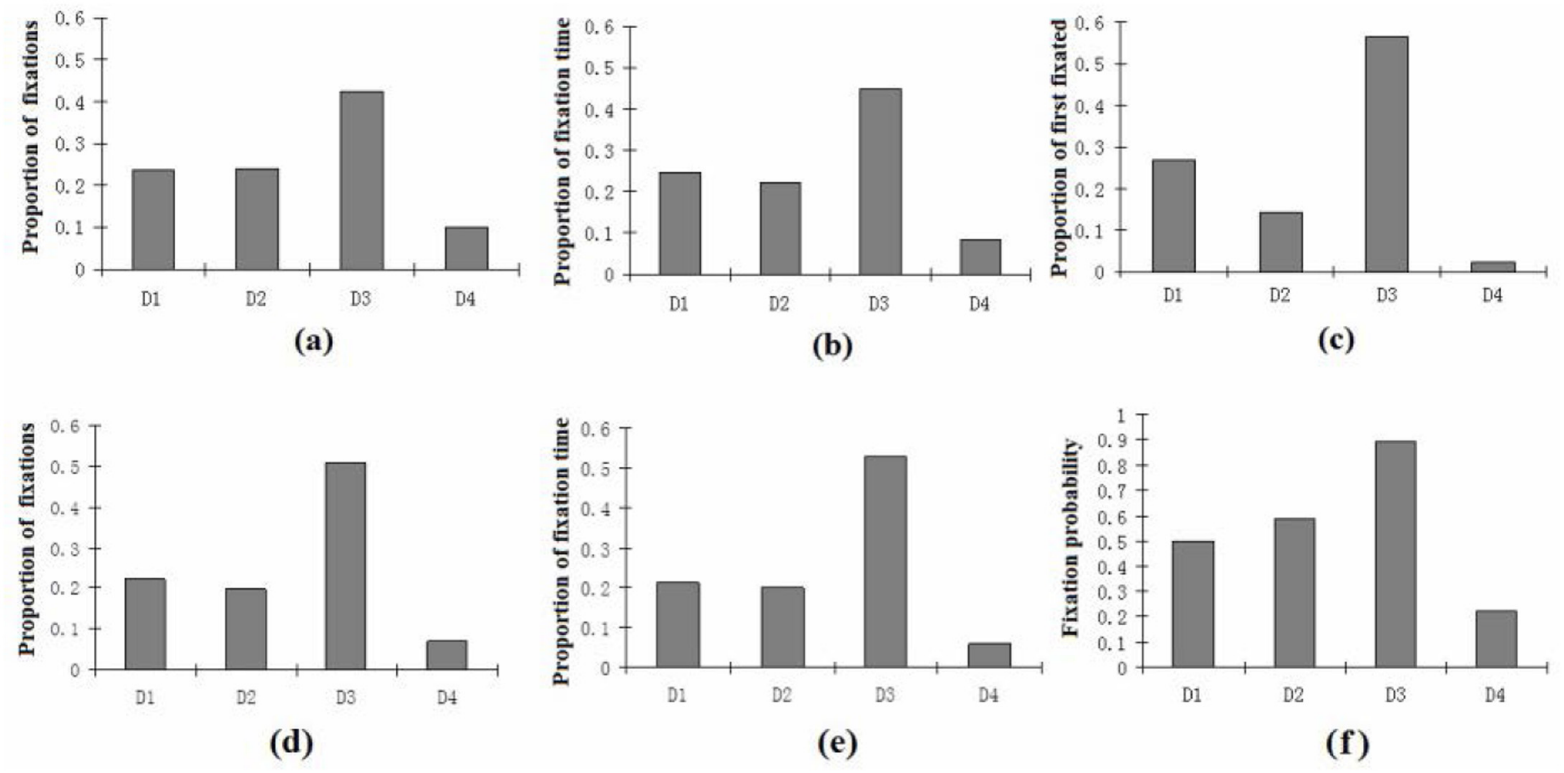
Eyetracking measures by dimensions, during Training (top, a-c) and Transfer (bottom, d-f). Proportion of fixations (a: Training phase; d: Transfer phase) is the proportion that the dimension was fixated across all fixations, regardless of trial. Proportion of fixation time (b: Training phase; e: Transfer phase) is the proportion of the total fixation time that the dimension was fixated, regardless of trial. Proportion of first fixation (c: data shown for Training only) is the proportion of all the first fixations of each trial that the dimension was fixated. Fixation probability (f: data is shown here for Transfer only; see Fig 3 for Training data) is the likelihood that the dimension was fixated at least once during each trial.

We then calculated the proportion of total fixations and total viewing time devoted to each dimension. As shown in Fig 2a and 2b, this analysis revealed a similar pattern as in Table 2, with D3 receiving almost half the fixations and fixation time, and D4 less than 10%, with D1 and D2 intermediate.

**Fig 3:**
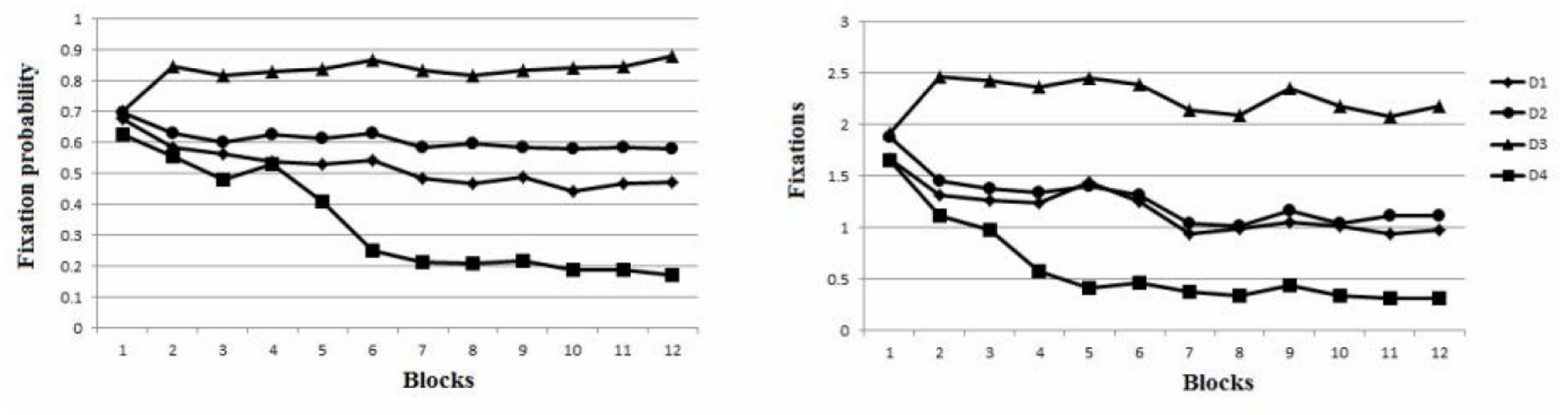
Fixations to each dimension across blocks. (a) Fixation probability: the likelihood that the dimension was fixated at least once during each trial. (b) Average total fixations per trial, including multiple fixations within a trial.

Having established that the fixation weightings reflected our predictions in the task overall, we examined how these weightings developed across training. Fig 3 shows the number of fixations, and fixation probability on each trial for each dimension across blocks of 10 trials. To construct Fig 3, we plotted performance through the 12th block, which was the mean number of blocks required by the subjects to meet the criterion. Subjects who reached criterion earlier than the 12^th^ block do not contribute to the mean data on subsequent blocks. Fig 3 indicates that during the first block all four dimensions were fixated on approximately equally often and were equally likely to be fixated on each trial. Differences between dimensions were apparent beginning in the second block. Fixation on D3 increased above the level devoted to the other dimensions beginning in the second block, both in terms of total fixations, and probability of being fixated at least once on each trial. During blocks 2 and 3, fixation of D1, D2, and D4 were similar, but beginning in block 4 the total number of fixations on D4 began to drop below the level of D2 and D1; beginning on blocks 5 and 6, the probability that D4 was fixated at all during a trial began to drop. D1 and D2 received similar number of total fixations across all blocks, but differed in terms of probability of at least one fixation on each trial, with D2 being more likely than D1.

### Transfer task data analyses

**Fig 4:**
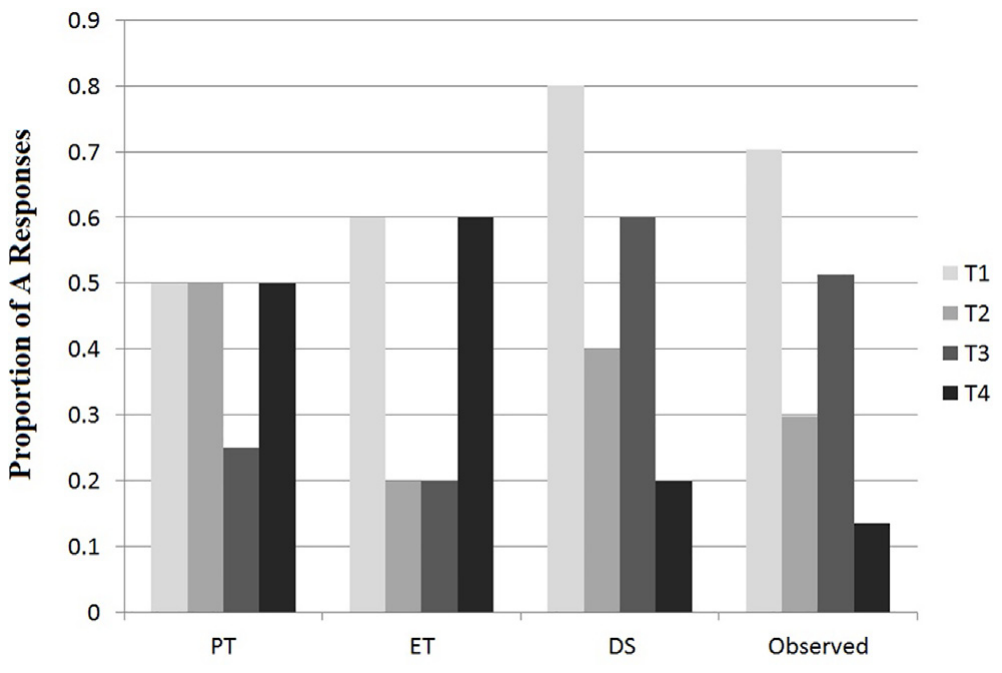
Observed data and prediction of PT, ET, and DS for likelihood of classifying the item as category “A” for each of the four transfer items. See Table 1 and Fig 1 for a description of each transfer item.

We calculated the proportion of A responses for each transfer item across subjects, as shown in Fig 4, observed data. Overall, T1 was usually categorized as A, T2 and T4 as B, and T3 approximately equally often as each category. A Wilcoxon signed ranks test was conducted to compare pairs of the transfer items: T1 and T3 (*z* = 2.53, *p* < .05); T3 and T2 (*t* = 1.46, *p*>.05); and T2 and T4 (*t* = 2.33, *p* < .05). Fig 4 illustrates these observed results alongside the predicted results from the exemplar theory (ET bars), prototype theory (PT bars), and dimensional sort (DS bars).

The rationale for the predicted results from the exemplar, prototype and dimensional sort strategies was derived as follows. Exemplar theory states that stimuli are categorized based on similarity to specific learned exemplars. Transfer items T2 and T3 are highly similar (match on 3 out of 4 dimensions) to individual members of category B (T2 is similar to B1, and T3 is similar to B3), but they are not similar to any of the members of category A. As a consequence, T2 and T3 should be classified into category B. Similarly, exemplar theory implies that T1 and T4 should be classified into category A (T1 is similar to A3, and T4 is similar to A1). In prototype theory, categorization is based on similarity to the prototype of each category. Two of the transfer items (T3 and T4) are equally similar to both the A and B prototypes. As a consequence, subjects should categorize these two items as category A or B equally often. T1 (*1212*) shares two features with the A prototype (*1311*), but only one with the B prototype (2322), so subjects should categorize it as A more often than as B; transfer item T2 (*1122*) shares two features as the B prototype but only one with the A prototype, so subjects should categorize it as B more often than A. Both simple exemplar and prototype theories assume that all features will be evaluated and contribute to the categorization decision. In contrast, dimensional sort proposes that subjects will evaluate the dimensions of each category in order of utility, based on the uniqueness and prototype strength of the features within each dimension: [D3, D1 or D2, D4]. As a result, we assessed overall match of the stimulus to the learned exemplars with greater weight for the earlier dimensions in the sequence. For example, dimensional sort categorizes T1 into category A because it matches the unique and prototypical feature in D3 within category A on D3, and the highly prototypical feature in D1 within category A.

### Mathematical model fit analyses

To enhance comparability of the current study with previous research, we used the methods developed by Rehder and Hoffman [5] to explore whether the prototype or exemplar model provides a better account of categorization during learning. We fit a five-parameter version of the GCM and the MPM to each subject’s learning data following the equations detailed in Rehder and Hoffman [5]. The only change from their method was that we used a genetic algorithm approach using matlab code developed in our laboratory to determine the degree of fit of each model for each subject. We used the sum of squared deviations (*SSD*) as our goodness-of-fit measure, in which smaller values indicate a better fit. Overall, the GCM was quantitatively superior to the MPM. The average *SSD* for the MPM was statistically worse (*M* = .96, *SD* = .50) than for the GCM [(*M* = .91, *SD* = .51), *t* (41) = 2.286, *p* < .05]. Of the 42 subjects, the majority (27, or 64%) was better fit by the GCM, and only 1 (3, or 2%) was better fit by the MPM. For the remaining subjects both models fit equally well.

Table 3 shows the attention weights based on the GCM and MPM models for each of the four dimensions. A test of within-subjects effects performed separately on the GCM and MPM dimension weights indicated a significant difference [*F* (37,3) = 7.652, *p* < .001] for the GCM weights and for the MPM weights [*F*(37,3) = 25.576, *p* < .001]; pairwise comparisons showed that the sequences of highest to lowest weighs for the dimensions were D3>D2 = D1>D4 for GCM [*t*(36) = 2.294, *p* < .05; *t*(36) = .033, *p*>.05; *t*(36) = 1.918, *p* = 0.63] and D2>D1 = D3 = D4 for MPM [*t*(36) = 5.933, *p* < .001; *t*(36) = .434, *p*>.05; *t*(36) = .486, *p*>.05]. Qualitatively, the weights from the GCM match the eyetracking results better than the weights from the MPM: both eyetracking and GCM attribute highest weight / most attention to D3, and least to D4, whereas MPM predicts that D2 should have the highest attentional weighting, yet eyetracking results showed D2 was fixated less than D3. Rehder and Hoffman [5] also found that attention weights measured by the eye tracker were similar to the weights derived in the exemplar-based GCM but not the prototype-based MPM.

**Table 3:** Attention weight based on GCM and MPM model averaged across the fits to the observed individual subject data.

Dimensions (D _k_)	D1	D2	D3	D4
GCM (W _k_)	**0.196**	**0.226**	**0.507**	**0.071**
MPM (W _k_)	**0.142**	**0.659**	**0.113**	**0.082**

### Fixation order and individual differences analyses

We hypothesized that subjects will not only weight some dimensions more highly than others, but that subjects will evaluate dimensions in order of utility. We argued that D3 would be evaluated first because it had both a unique and highly typical feature. To examine fixation order, we identified for each trial the first dimension that received a fixation. As shown in Fig 2f, across subjects and trials, D3 was fixated first more than half the time, and D4 was almost never fixated first.

Although overall subjects fixated on D3 first, there was variability across subjects. Of the 42 subjects, 23 fixated D3 first on at least 60% of the trials. However, 9 subjects fixated on D1 first on more than 60% of the trials, and 9 subjects did not fixate first on any single dimension on more than 60% of the trials. We examined whether these differences in initial fixation choice were related to other measures of learning, including number of blocks to criterion, and how well the subject’s individual data was fit by the GCM and MPM models, indicating a stronger reliance on exemplar and prototype strategies, respectively. We found that the three groups did not differ in blocks to criterion, indicating that all three patterns of initial fixation may be equally compatible with successful learning. There was a significant effect of group on GCM and trend toward a significant effect on MPM model fits, *F* (42, 2) = 3.443, *p*<0.05 and *F* (42, 2) = 2.784, *p* = 0.074, respectively. In both cases, post hoc tests showed that subjects who fixated D1 first were fit better. Subjects who view D1 first may be less likely to use a rule-based strategy focusing on individual dimensions, and more likely to evaluate stimuli via strategies in which the stimulus is evaluated as a whole, as is the case for both exemplar and prototype strategies.

## Discussion

How do people learn a new category and categorize new stimuli? In this research, we adopted a multiple-value-feature category structure and used eye-tracking technology to assess dimension weighting during category learning. There are two primary findings supporting our argument that feature uniqueness affects dimension weighting beyond the degree of typicality. First, D3, a unique and strongly typical dimension, received more fixations and longer observation times than strongly typical dimension D1, although both were equated for typicality. Second, performance on the transfer items was also consistent with strong differential weighting of D3 with respect to D1 and D2. This differential weighting of D3 can be explained rule based strategies but not by basic exemplar or prototype theories. However, a more sophisticated computational implementation of an exemplar-based theory, the GCM, provided a better fit for the behavior and eye-tracking data than a computational model of prototype theories, the MPM.

### Weighting dimensions: Typicality and uniqueness

This study used both eyetracking and mathematical modeling methods and indicated that a feature’s typicality and uniqueness were two major factors that determined the weight of each dimension. D3 and D1 were equated for typicality, but differed in uniqueness; the stronger weighting of D3 indicates an additional role for uniqueness beyond typicality alone. However, it is less clear how subjects trade off between typicality and uniqueness. D1 was strongly prototypical but not unique, whereas D2 had a unique feature that was only weakly prototypical. If unique cues are valued regardless of prototype strength, we would predict more attention to D2, whereas if probabilistic strength of the stimuli is valued then we would predict more attention to D1. We found overall that the two were fixated equally often, and had similar mean fixation times. Intriguingly, we found that although D1 and D2 received the same number of total fixations, D2 was more likely to be fixated at least once on every trial in a block than D1. This pattern of results indicates that subjects were more consistent about fixating D2 regularly across trials. In contrast, D1 was ignored on a higher proportion of trials, but on trials that it was fixated it received a higher number of multiple fixations. This pattern may indicate that the information in D1 received more attention on trials where it was potentially relevant, and ignored on trials where it was not relevant.

Unique features are similar to what Chin-Parker and Ross [7] termed diagnostic features, defined as features that are useful for determining category membership. They manipulated diagnosticity on the basis of overlap of the prototypes used; features common to both prototypes are highly typical of both categories, but not diagnostic of either category, whereas features that differed across prototypes are diagnostic regardless of degree of typicality across studied exemplars. However in their study diagnosticity was probabilistic and not deterministic; a single diagnostic feature did not on its own give sufficient information for categorization. In our study, unique features when present gave deterministic information about the correct category membership for the item.

### Ordered search across dimensions

We hypothesized that in a multidimensional task subjects may determine category membership by sequentially evaluating feature dimensions rather than assessing all dimensions on the basis of similarity to a prototype or previously studied exemplars. For the task we used, we argued that subjects would first evaluate D3, which contained a unique and prototypical feature that could be used to determine category membership for 6 / 10 stimuli, then would evaluate D1 and D2, and would finally evaluate D4. Our eyetracking measures were consistent with these predictions, with D3 fixated first by most subjects on most trials, and receiving the highest number of fixations overall. Our transfer task results were also consistent with this ordering and weighting, with subjects overall categorizing the stimuli primarily but not exclusively in accordance with match on D3, and to a lesser degree on the basis of match with D1 and D2. Our results are consistent with work by Blair, Chen, and colleagues [18,19] who found that over time subjects primarily fixated on relevant features and did not fixate on irrelevant features. These previous studies also found differences in order of fixation, with highly relevant features fixated earlier in the trial, consistent with our finding of D3 being fixated first by a high proportion of subjects.

Another question is whether subjects always evaluated all dimensions, or whether subjects evaluated only as many dimensions as are necessary for determining category membership. Our results support the latter. D3 was fixated on almost every trial, but the other dimensions were fixated much less reliably. Late in learning the likelihood of viewing D4 drops to 10%, and D1 and D2 are only viewed on half the trials. This pattern argues against exhaustive search and supports the idea that subjects terminate evaluation as soon as possible. These results are consistent with Meier and Blair [9] who found that subjects maximized efficiency of eye movements, even to the extent of choosing as an initial fixation a feature that began an efficient search path rather than choosing the most informative feature overall.

We argued that dimensions would be evaluated and weighted in order. This can be seen as a rule-based theory consisting of a series of rules for each dimension that together result in determining category membership. The general idea that people may use rules as a basis for classification is supported by a number of studies [11–13, 20–23]. For example, Fific, Little, and colleagues have developed a logical rule model [24–26] to predict response-time (RT) data from subjects trained to classify integral-dimension color stimuli into rule-based categories. This model suggests that people make independent decisions about the locations of stimuli along a set of component dimensions.

We hypothesized that dimensions would be evaluated in order of utility. This is similar to strategies developed to account for decision making, sometimes termed lexicographic strategies or heuristics. One such heuristic is called the “Take The Best” (TTB) heuristic [27, 28]; it proposes that cues are ranked in terms of utility or validity, and the decision maker evaluates cues in order, stopping as soon as a cue is found that can be used to discriminate between the options. It has its roots in the classic studies of Tversky [29], who proposed that intransitivity during decision making could be due to use of a similar strategy. TTB does not require that the subject assess and integrate across all cue values, and thus will fail if a combination of less valid cues would in fact result in a more optimal decision. However, it has been shown to outperform strategies that do integrate, particularly in situation where there are insufficient resources for the more demanding integrative strategies [30]. Several recent studies have argued that subjects may be able to flexibly switch between non-integrative and integrative strategies depending on contextual factors [31,32]. In the domain of categorization, Juslin [33] found that subjects will use an integrative strategy if it significantly improves accuracy, but will fall back on the simpler strategy if accuracy levels are similar. In our study, subjects primarily weighted D3, but did not solely rely on it, indicating some level of integration.

An open question about dimensional search and similar decision making tasks is whether the same type of explicit or implicit representation underlies all stages of the decision process. Explicit rules are those that typically can be verbalized by subjects, and recruit neural systems underlying working memory and declarative memory [11, 34]. One possibility is that subjects develop a complete series of verbalizable rules for all dimensions. However, as the number of dimensions increases, the working memory load is likely to increase past the typical capacity of subjects. At the other extreme, it is possible that categorization in this task is implicit in the sense that subjects would not be able to verbally describe the strategy used for categorization. Finally, an intermediate possibility would be that subjects gain verbalizable knowledge of one or two dimensions, and after evaluating these dimension use non-verbalizable evaluation strategies on the remaining dimensions.

## Supporting Information

S1 Data Three files zip to a file of rar format; rawall.xls is a raw data of eyetracking; fixation.sav is a SPSS data file made from rawall.xls; beh.sav is a behavor data file. (RAR)
